# Tomato Leaf Curl New Delhi Virus Spain Strain Is Not Transmitted by *Trialeurodes vaporariorum* and Is Inefficiently Transmitted by *Bemisia tabaci* Mediterranean between Zucchini and the Wild Cucurbit *Ecballium elaterium*

**DOI:** 10.3390/insects14040384

**Published:** 2023-04-15

**Authors:** Alessia Farina, Carmelo Rapisarda, Elvira Fiallo-Olivé, Jesús Navas-Castillo

**Affiliations:** 1 Instituto de Hortofruticultura Subtropical y Mediterránea “La Mayora” (IHSM-UMA-CSIC), Consejo Superior de Investigaciones Científicas, 29750 Algarrobo-Costa, Spain; alessia.farina@phd.unict.it (A.F.); efiallo@eelm.csic.es (E.F.-O.); 2 Applied Entomology Section, Department of Agriculture, Food and Environment (Di3A), University of Catania, 95123 Catania, Italy; carmelo.rapisarda@unict.it

**Keywords:** *Begomovirus*, *Geminiviridae*, tomato leaf curl New Delhi virus, transmission, whiteflies, *Bemisia tabaci*, *Trialeurodes vaporariorum*, cucurbits, *Ecballium elaterium*, wild plants

## Abstract

**Simple Summary:**

Begomoviruses constitute a successful group of emerging plant viruses threatening vegetable, root and fiber crops worldwide that are transmitted in nature by whiteflies of the *Bemisia tabaci* complex. Tomato leaf curl New Delhi virus is a paradigmatic example of a begomovirus that has recently emerged in Mediterranean countries after movement from its original location in the Indian subcontinent. The Mediterranean isolates of this virus belong to a novel strain, named “Spain strain”, which infects zucchini and other cucurbits but is poorly adapted to tomato. This work aimed to clarify some aspects of the whitefly transmission of tomato leaf curl New Delhi virus. It was shown that contrary to a recent study reporting the transmission of an Indian isolate of the virus by the greenhouse whitefly (*Trialeurodes vaporariorum*), the Mediterranean isolate is not transmitted by this insect. In addition, the most prevalent *Bemisia tabaci* species, Mediterranean, is not an efficient vector of this begomovirus between zucchini plants and the wild cucurbit *Ecballium elaterium*. These results suggest that this wild plant, although frequently infected, may not play a relevant role as a reservoir in the epidemiology of the disease caused by tomato leaf curl New Delhi virus Spain strain.

**Abstract:**

Tomato leaf curl New Delhi virus (ToLCNDV) is a bipartite begomovirus (genus *Begomovirus*, family *Geminiviridae*) persistently transmitted, as with all other begomoviruses, by whiteflies (Hemiptera: Aleyrodidae) of the *Bemisia tabaci* cryptic species complex. The virus, originally from the Indian subcontinent, was recently introduced in the Mediterranean basin, where it is currently a major concern for protected and open-field horticulture. The Mediterranean ToLCNDV isolates belong to a novel strain named “Spain strain” (ToLCNDV-ES), which infects zucchini and other cucurbit crops but is poorly adapted to tomato. Recently, it has been reported that another whitefly, *Trialeurodes vaporariorum*, is able to transmit an isolate of ToLCNDV from India which infects the chayote plant, a cucurbit. The present work aimed to clarify some aspects of whitefly transmission of ToLCNDV-ES. It was shown that *T. vaporariorum* is not able to transmit ToLCNDV-ES between zucchini plants. In addition, *Ecballium elaterium* may not act as a relevant reservoir for this virus strain in the Mediterranean basin, as *B. tabaci* Mediterranean (MED), the most prevalent species of the complex in the region, is not an efficient vector of this begomovirus between cultivated zucchini and wild *E. elaterium* plants.

## 1. Introduction

A high number of emerging diseases affecting vegetable, root and fiber crops worldwide are caused by viruses transmitted by sap-sucking insects, mainly hemipterans, including aphids, leafhoppers, planthoppers and whiteflies. Most of these viruses belong to the genus *Begomovirus* (family *Geminiviridae*), the largest genus in the entire virosphere, with 409 recognized species [1]. Begomoviruses constitute an extremely successful group of emerging plant viruses, present in both the Old and New World [2]. Indeed, the two major phylogenetic groups within the genus are related to geographical origin: Old World (OW) and New World (NW) begomoviruses; the others being the legumoviruses and the sweepoviruses. Begomoviruses are transmitted in a persistent mode by adults of the whitefly (Hemiptera: Aleyrodidae) *Bemisia tabaci* (Gennadius) sensu lato, which is a complex of more than 40 cryptic, morphologically indistinguishable species [3,4]. The transmission of a begomovirus, tomato yellow leaf curl virus, has been shown to be propagative, with the virus being replicated in the salivary glands of the vector [5].

Genomes of begomoviruses are either bipartite or monopartite [1]. The genomes of bipartite begomoviruses are composed of two components of circular single-stranded DNA known as DNA-A and DNA-B, each 2.5–2.7 kb in size. Open reading frames are organized from an intergenic region (IR) that includes the origin of replication. Within the IR, a segment of approximately 200 nt (common region) is almost identical between the two components of each bipartite begomovirus. The DNA-A virion-sense strand encodes two proteins: the coat protein and the AV2 protein, which is involved in virus movement. Bipartite begomoviruses from the New World lack the AV2 ORF. The DNA-A complementary-sense strand encodes four proteins: the replication-associated protein, a transcriptional activator protein, a replication enhancer protein and the AC4 protein. DNA-B encodes two proteins: one on the virion-sense strand, a nuclear shuttle protein, and one on the complementary-sense strand, which is a movement protein. Genomes of monopartite begomoviruses are composed of a single component, analogous to the DNA-A of bipartite begomoviruses. Genomic components of begomoviruses are separately encapsidated within twinned (geminated) quasi-icosahedral virions [1].

One of the most damaging and widespread OW begomoviruses is tomato leaf curl New Delhi virus (ToLCNDV), a typical bipartite begomovirus. ToLCNDV was initially found infecting tomato (*Solanum lycopersicum* L.) crops in India [6] and is currently a major concern to horticulture in many regions of the world [7,8]. In addition to important solanaceous (eggplant, pepper, potato and tomato) and cucurbit (cucumber, melon, pumpkin and zucchini) crops, ToLCNDV has been shown to infect a wide host range of dicot plants, both crops and wild plants, belonging to the families Acanthaceae, Amaranthaceae, Apiaceae, Apocynaceae, Asteraceae, Caricaceae, Convolvulaceae, Euphorbiaceae, Fabaceae, Malvaceae, Oleaceae, Papaveraceae and Polygonaceae [8]. Symptoms caused by typical Indian ToLCNDV isolates in tomato include stunting, leaf curling and deformation, interveinal yellowing and yield losses.

ToLCNDV was limited to the Indian subcontinent and other Asian countries until the 2012/2013 season, when it was first detected in the Mediterranean basin infecting zucchini (*Cucurbita pepo* L.) crops in the southeastern provinces of continental Spain, Murcia and Almería [9]. Symptoms observed in infected zucchini plants included curling, vein swelling and severe mosaic in young leaves as well as short internodes and fruit skin roughness [9]. In the following years, the virus caused serious epidemic outbreaks in cucurbit crops in this area, both in greenhouses and open fields [10,11]. ToLCNDV was soon detected infecting zucchini crops in other countries in southern Europe and North Africa, including Tunisia (2015) [12], Italy (2015) [13], Morocco (2017) [14], Algeria (2018) [15] and Greece (2018) [16]. The genetic analysis of the viral isolates present in Spain, closely related to those characterized in other Mediterranean countries, showed that they all belong to a novel strain, named “Spain strain” (ToLCNDV-ES) [10]. Although ToLCNDV-ES isolates have been detected infecting tomato plants in Spain in the field, these infections appeared to be anecdotal [11,17,18]. Furthermore, agroinoculation experiments have confirmed that isolates of this strain are very efficient at infecting cucurbits but seem to be poorly adapted to infect tomato: in an experiment carried out in Spain, 20 out of 20 zucchini plants were infected while only 1 out of 20 tomato plants was [10].

Several wild plant species have been reported to be naturally infected by ToLCNDV-ES in Spain: the cucurbit *Ecballium elaterium* (L.) A. Rich., the solanaceous *Datura stramonium* L. and *Solanum nigrum* L. and the asteraceous *Sonchus oleraceus* L. [11], the most commonly found being *E. elaterium*. This suggests a relevant role for this perennial wild cucurbit in ToLCNDV epidemiology in the Mediterranean basin by acting as a source of inoculum. ToLCNDV, as with all begomoviruses, is transmitted in a persistent manner by whiteflies of the *B. tabaci* cryptic species complex [2]. Surprisingly, however, it has been reported that another whitefly species, *Trialeurodes vaporariorum* (Westwood), the greenhouse whitefly, was able to transmit a ToLCNDV isolate found infecting chayote [*Sechium edule* (Jacq.) Sw.], a cucurbit, in India [19].

In the absence of direct control measures, management of plant virus diseases relies on a profound knowledge of the interactions established between the virus, the host plant and the environment. In the case of viruses transmitted by a biological vector, e.g., insects, the complexity of the system increases and the role of vectors takes on special importance. In line with this, the present work aimed to assess some aspects of ToLCNDV-ES transmission by whiteflies. A first objective was to determine whether ToLCNDV-ES is transmissible by the whitefly *T. vaporariorum*. Secondly, considering that the cucurbit *E. elaterium* is frequently infected by ToLCNDV-ES in Spain, the transmissibility of this virus by *B. tabaci* Mediterranean (MED) species between this wild plant and zucchini plants was also evaluated.

## 2. Materials and Methods

### 2.1. Virus Isolate and Plant Agroinoculation

Dimeric infectious clones for DNA-A and DNA-B genomic components of ToLCNDV-ES, isolate RV1 (E. Fiallo-Olivé and J. Navas-Castillo, in preparation) were used in *Agrobacterium tumefaciens*-mediated inoculation (agroinoculation). For agroinoculation assays, liquid cultures of *A. tumefaciens* strain C58C1 harboring each dimeric construct were added at 1:1000 to YEP liquid media containing kanamycin (50 µg mL^−1^) and rifampicin (50 µg mL^−1^) and grown at 28 °C for 2 d. Cultures were centrifuged at 3100× *g* for 20 min at room temperature and then resuspended in 10 mM 2-(*N*-morpholino)ethanesulfonic acid (MES) (pH 5.6), 10 mM MgCl_2_ and 150 M acetosyringone to a final OD_600_ of 1.0. Equal volumes of DNA-A and DNA-B cultures were mixed before stem puncture inoculation of zucchini cv. Milenio (Semillas Fitó, Barcelona, Spain) or *E. elaterium* plants. Agroinoculated plants were maintained in an insect-proof growth chamber (25 °C during the day and 18 °C at night, 70% relative humidity, with a 16 h photoperiod at 250 µmol s^−1^ m^−2^ photosynthetically active radiation) for symptom development and further analysis.

### 2.2. Virus Detection

At 30 d post-inoculation (dpi), apical leaves were used for tissue blots of petiole cross sections (tissue printing) performed on positively charged nylon membranes (Roche Diagnostics, Mannheim, Germany) and hybridization using digoxigenin-labelled DNA probes specific to DNA-A and DNA-B of ToLCNDV-ES prepared by polymerase chain reaction according to the DIG-labelling detection kit (Roche Diagnostics, Mannheim, Germany) as previously described [10]. Hybridization analysis of tissue blots was performed at high stringency conditions (hybridization at 65 °C followed by washing steps with 0.1× saline sodium citrate, 0.1 sodium dodecyl sulfate at 65 °C). Hybridization signals were detected on X-ray film after treatment with CDP-Star (Roche Diagnostics, Mannheim, Germany) and developed following a conventional photographic process.

### 2.3. Whitefly Transmission

Nonviruliferous *B. tabaci* Mediterranean (MED, formerly biotype Q) and *T. vaporariorum* populations were reared on *Solanum muricatum* Aiton cv. Sweet Long and *Nicotiana glauca* Graham plants, respectively, in insect-proof BugDorm cages (MegaView Science, Taichung, Taiwan). Unsexed adult whiteflies were collected with an aspirator and transferred to clip-cages (50 insects per clip-cage) on zucchini cv. Milenio or *E. elaterium* plants infected with ToLCNDV-ES isolate RV1. Whitefly-inoculated plants were maintained in BugDorm cages inside a growth chamber (25 °C during the day and 18 °C at night, 70% relative humidity, with a 16 h photoperiod at 250 µmol s^−1^ m^−2^ photosynthetically active radiation). After an acquisition-access period (AAP) of 48 h, the clip-cages containing the whiteflies were transferred onto healthy zucchini or *E. elaterium* plants at the two-leaf stage. Following an inoculation-access period (IAP) of 48 h, clip-cages were detached from the plants that were treated with insecticides (Confidor 20 LS, 20% imidacloprid and Atominal 10 EC, 10% pyriproxyfen). Four source plant–target plant–whitefly species combinations were assayed as follows: (i) zucchini–zucchini (*n* = 20)–*T. vaporariorum*, (ii) zucchini–zucchini (*n* = 20)–*B. tabaci* MED, (iii) zucchini–*E. elaterium* (*n* = 10)–*B. tabaci* MED, and (iv) *E. elaterium–*zucchini (*n* = 10)–*B. tabaci* MED. Two independent experiments were carried out per combination. Plants were examined daily for the presence of symptoms. At 30 dpi, an apical leaf per plant was utilized for tissue printing and molecular hybridization using digoxigenin-labelled DNA probes specific to the ToLCNDV-ES DNA-A and DNA-B components as described above.

## 3. Results and Discussion

### 3.1. ToLCNDV-ES Is Not Transmitted by T. vaporariorum between Zucchini Plants

**Figure 1:**
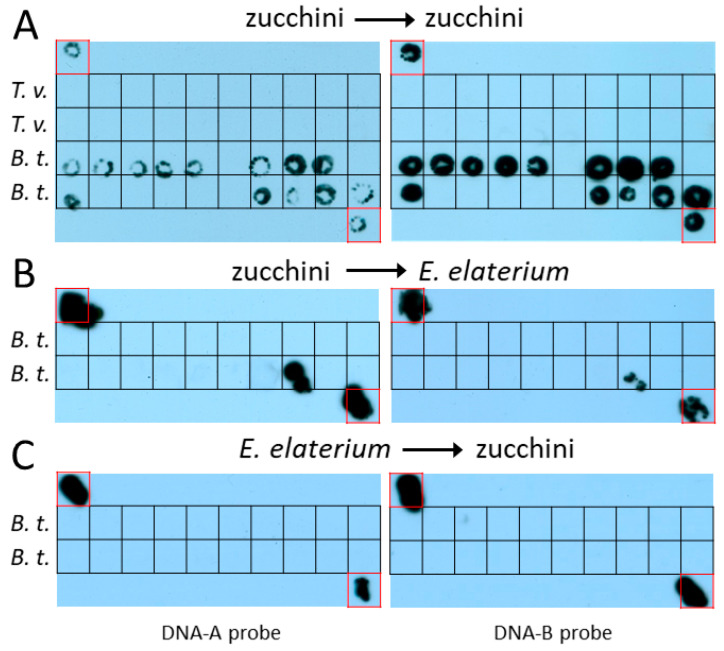
Agroinoculation experiments with tomato leaf curl New Delhi virus Spain strain using different source plant–target plant–whitefly species combinations. ToLCNDV (DNA-A and DNA-B) was detected by molecular hybridization after tissue printing on nylon membranes using specific digoxigenin-labelled DNA probes for each viral component. (**A**) Prints of zucchini plants inoculated using *Trialeurodes vaporariorum* (*T. v.*) or *B. tabaci* MED (*B. t.*) as a vector and infected zucchini plants as the virus source. (**B**) Prints of *Ecballium elaterium* plants using *B. t.* as a vector and infected zucchini plants as the virus source. (**C**) Prints of zucchini plants using *B. t.* as a vector and infected *E. elaterium* plants as the virus source. Blots from one of the two independent experiments (Exp. 1 in Table 1) carried out for each source plant–target plant–whitefly species combination are shown. Prints of ToLCNDV-infected zucchini plants included as positive controls are outlined with red squares.

In the transmission assays with *T. vaporariorum* using ToLCNDV-ES-agroinfected zucchini plants both as the source of inoculum and target plants, none of the 40 inoculated zucchini plants (two independent experiments with 20 plants each) were infected by the virus (Table 1). However, when *B. tabaci* MED was used, 13 and 11 out of 20 target zucchini plants were infected in two independent experiments, respectively (Table 1). Infection was revealed by the expression of typical viral symptoms and confirmed by molecular hybridization using ToLCNDV-ES DNA-A and DNA-B probes (Figure 1A). These results contrast with data reported by Sangeetha et al. [19], who found successful transmission by *T. vaporariorum* of a ToLCNDV isolate from chayote in India. It must be taken into account that the differences observed between that report and the current work might be due to differences between virus isolates, *T. vaporariorum* genotypes, plant hosts or even experimental conditions. In this regard, it is worth mentioning that the experiments carried out with ToLCNDV-ES used different numbers of whitefly adults per inoculated plant (50 vs. 20) and AAP/IAP times (48/48 h vs. 4/24 h) that the experiment reported as successful for ToLCNDV-chayote. The fact that transmission by *T. vaporariorum* was reported to occur only in the experiment carried out with an AAP of 4 h but not in those of 1, 8, 12 or 24 h [19] opens the possibility that an unconventional mechanism, yet to be identified, could be involved in the transmission of ToLCNDV-chayote by this whitefly [19].

*T. vaporariorum* is known to be a vector of plant viruses other than begomoviruses in a semi-persistent manner, such as some criniviruses (genus *Crinivirus*, family *Closteroviridae*) [20] and torradoviruses (genus *Torradovirus*, family *Secoviridae*) [21]. Moreover, some criniviruses (e.g., tomato chlorosis virus) and torradoviruses (e.g., tomato torrado virus) are transmitted by both *B. tabaci* and *T. vaporariorum* [22,23]. It should be stressed that *T. vaporariorum* is virtually absent in areas where ToLCNDV-ES has been found infecting *E. elaterium* and cultivated cucurbits in Spain. In any case, confirmation that ToLCNDV is transmitted by *T. vaporariorum* awaits further investigation.

### 3.2. ToLCNDV-ES Is Not Efficiently Transmitted by B. tabaci MED between Zucchini and E. elaterium

In the transmission assays with *B. tabaci* MED using ToLCNDV-ES-agroinfected zucchini plants as the source of inoculum and *E. elaterium* as target plants, only one out of the twenty *E. elaterium* plants used in two independent experiments were infected, as revealed by the absence of symptoms at 30 dpi and negative molecular hybridization results (Table 1) (Figure 1B). The frequent presence of ToLCNDV-ES-infected *E. elaterium* plants observed in the Spanish provinces of Murcia [11] and Málaga (our unpublished results) contrasts with the low transmission rate from zucchini to this wild cucurbit using *B. tabaci* MED, the most prevalent species of the complex in the region. On the other hand, none of the 20 zucchini plants inoculated with the same whitefly vector starting from infected *E. elaterium* plants in two independent experiments were infected (Table 1) (Figure 1C). This is reminiscent of the finding that pepper (*Capsicum annuum* L.) behaves as a dead-end for another begomovirus, tomato yellow leaf curl virus (TYLCV), in Spain [24]. The authors of that study found that TYLCV could not be transmitted from infected pepper plants, most likely because of the low viral titers present, suggesting that this crop acts as a severe bottleneck in the epidemiological cycle of this begomovirus. As was suggested for the TYLCV/pepper system [24], it cannot completely be ruled out that *E. elaterium* may serve as a low-efficiency reservoir when vector populations are extremely high. Also, *E. elaterium*, being a perennial plant, can become infected after successive whitefly colonization waves over the years even if the efficiency of infection by *B. tabaci* is low. 

In summary, our findings do not support ToLCNDV transmission by whiteflies other than members of the *B. tabaci* complex and provide insight into the questionable role of *E. elaterium*, a wild cucurbit plant, in ToLCNDV epidemics in the Mediterranean basin. These findings are a good starting point to advance our understanding of ToLCNDV epidemiology, thus helping to develop control strategies to fight the harmful disease that this virus causes to important crops.

## Figures and Tables

**Table 1:** Transmission experiments of tomato leaf curl New Delhi virus Spain strain between zucchini cv. Milenio and *Ecballium elaterium* plants by *Trialeurodes vaporariorum* and *Bemisia tabaci* MED.

ToLCNDV-ES-Infected Source Plant	Target Plant	Whitefly Species	No. Infected Plants/ No. Inoculated Plants *	
			Exp. 1	Exp. 2
Zucchini	Zucchini	*T. vaporariorum*	0/20	0/20
Zucchini	Zucchini	*B. tabaci* MED	13/20	11/20
Zucchini	*E. elaterium*	*B. tabaci* MED	1/10	0/10
*E. elaterium*	Zucchini	*B. tabaci* MED	0/10	0/10

* Infection was determined by tissue-printing and molecular hybridization with digoxigenin-labelled ToLCNDV DNA-A and DNA-B probes. Blots from experiment (Exp.) 1 of each source plant–target plant–whitefly species combination are shown in Figure 1.

## Data Availability

The data presented in this study are available in this article.
